# Supplementary material for: Star-Shaped Poly(2-ethyl-2-oxazoline) and Poly(2-isopropyl-2-oxazoline) with Central Thiacalix[4]Arene Fragments: Reduction and Stabilization of Silver Nanoparticles
Source: Polymers (Basel). 2019 Dec 4;11(12):2006. doi: 10.3390/polym11122006 (PMC6960741; doi:10.3390/polym11122006)
Supplement: Supplementary file 1 [file polymers-11-02006-s001.pdf]

polymers

MDPI

Supplementary Material

Star-Shaped Poly(2-ethyl-2-oxazoline) and Poly(2-isopropyl-2-oxazoline) with Central Thiacalix[4]Arene Fragments: Reduction and Stabilization of Silver Nanoparticles

Alexey Lezov [1], Alexander Gubarev [1], Maria Mikhailova [1], Alexandra Lezova [1], Nina Mikusheva [1], Vladimir Kalganov [1], Marina Dudkina [2], Andrey Ten'kovtsev [2], Tatyana Nekrasova [2], Larisa Andreeva [2], Natalia Saprykina [2], Ruslan Smyslov [2,3], Yulia Gorshkova [4], Dmitriy Romanov [5], Stephanie Höppener [6,7], Igor Perevyazko [1] and Nikolay Tsvetkov [1,*]

[1] Department of Molecular Biophysics and Polymer Physics, St. Petersburg State University, Universitetskaya nab., 7/9, St. Petersburg, 199034, Russian Federation
[2] Institute of Macromolecular Compounds of the Russian Academy of Sciences, Bolshoi pr 31, St. Petersburg, 199004, Russian Federation
[3] Institute of Biomedical Systems and Technologies, Peter the Great St. Petersburg Polytechnic University, Polytechnicheskaya 29, 195251 St. Petersburg, Russian Federation
[4] Joint Institute for Nuclear Research, Joliot-Curie 6, Dubna, Moscow region 141980, Russian Federation
[5] Institute of Silicate Chemistry of the Russian Academy of Sciences, Adm. Makarova emb. 2, 199034 St.Petersburg, Russia
[6] Laboratory of Organic and Macromolecular Chemistry (IOMC), Friedrich Schiller University Jena, Humboldt Straße 10, 07743 Jena, Germany
[7] Jena Center for Soft Matter (JCSM), Philosophenweg 7,07743 Jena, Germany
Correspondence: n.tsvetkov@spbu.ru or n.tsvetkov@mail.ru; Tel.: +7-812-428-7598

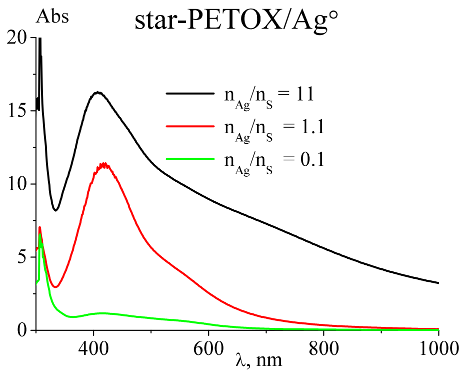

a

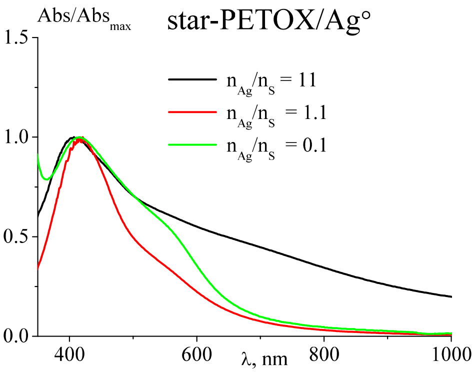

b

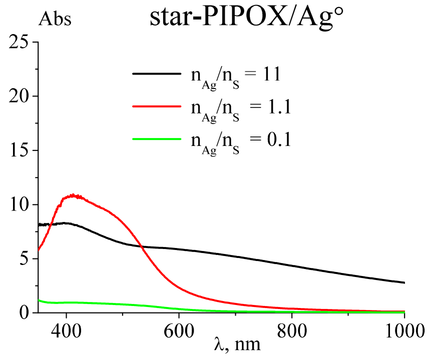

c

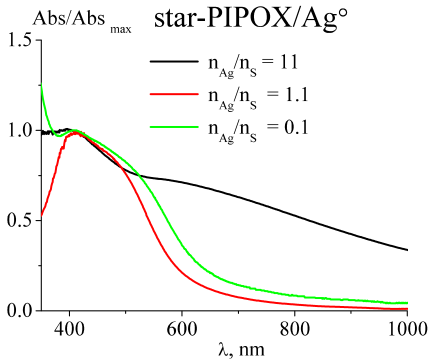

d

Figure S1. Absorption spectrums of star-PETOX/Ag^0 (**a**), star-PIPOX/Ag^0 (**b**); normalized absorption spectrums of star-PETOX/Ag^0 (**c**), star-PIPOX/Ag^0 (**d**).

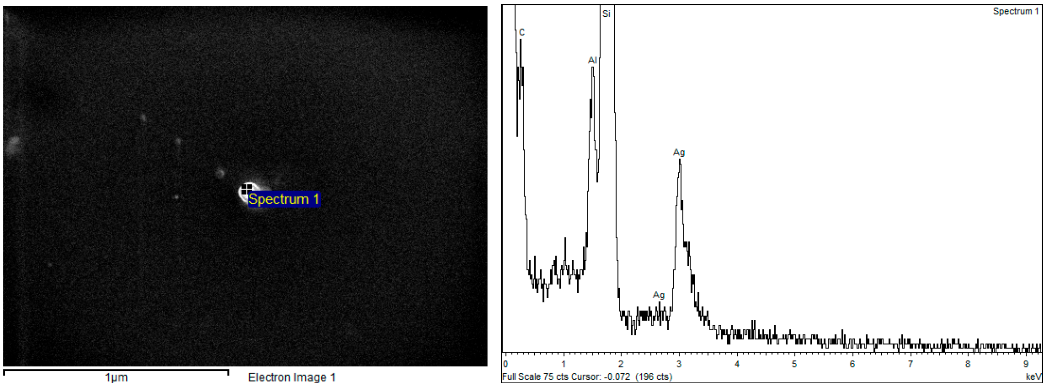

a

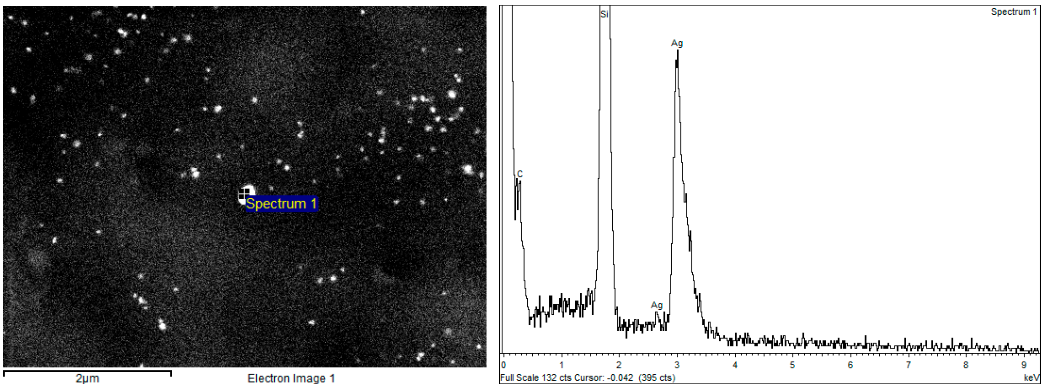

b

Figure S2. SEM image and corresponding energy-dispersive X-ray spectra for complexes of silver nanoparticles with star-PETOX (**a**) and star-PIPOX (**b**) macromolecules.

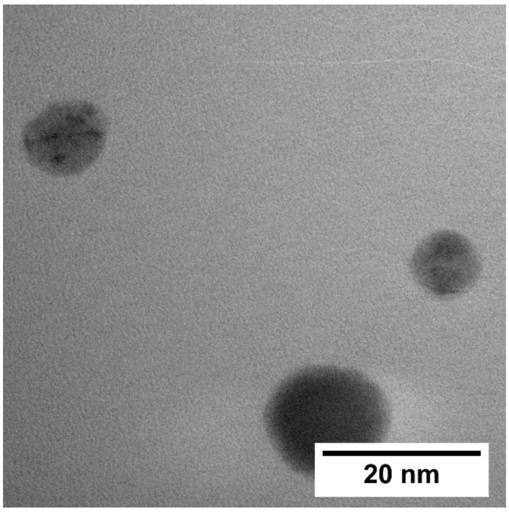

(**a**)

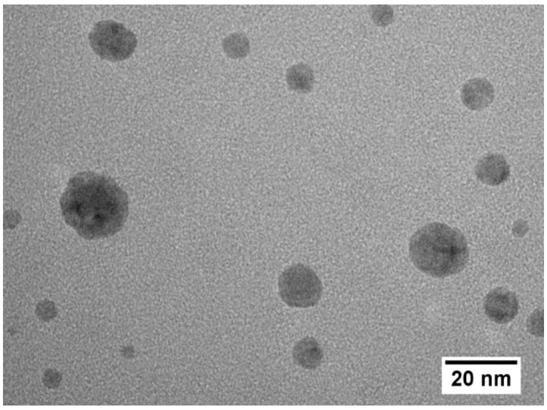

(**b**)

Figure S3. (**a**). High resolution TEM of star-PETOX/Ag^0 nanoparticles. (**b**). High resolution TEM of star-PIPOX/Ag^0 nanoparticles.

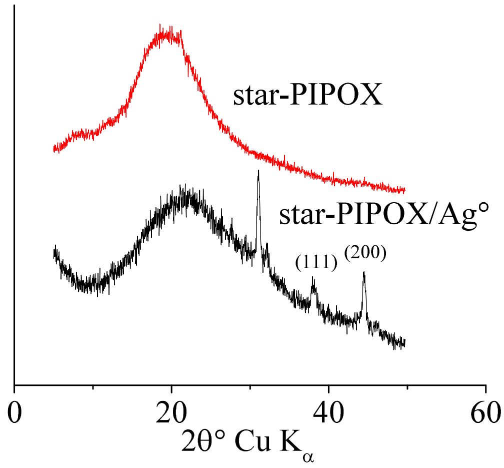

Figure 4. Diffractograms of star-PIPOX and star-PIPOX/Ag^0 powder-like samples.

AUC data.

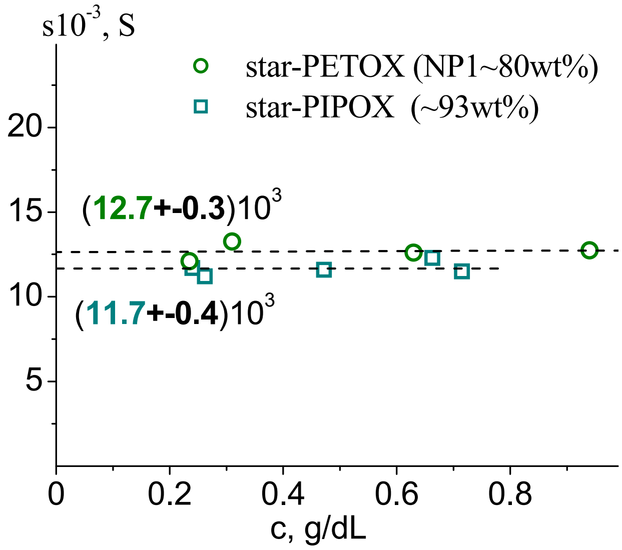

Figure S5. The concentration dependences of sedimentation coefficients (s) obtained for the maijor fraction of stabilized Ag NP.

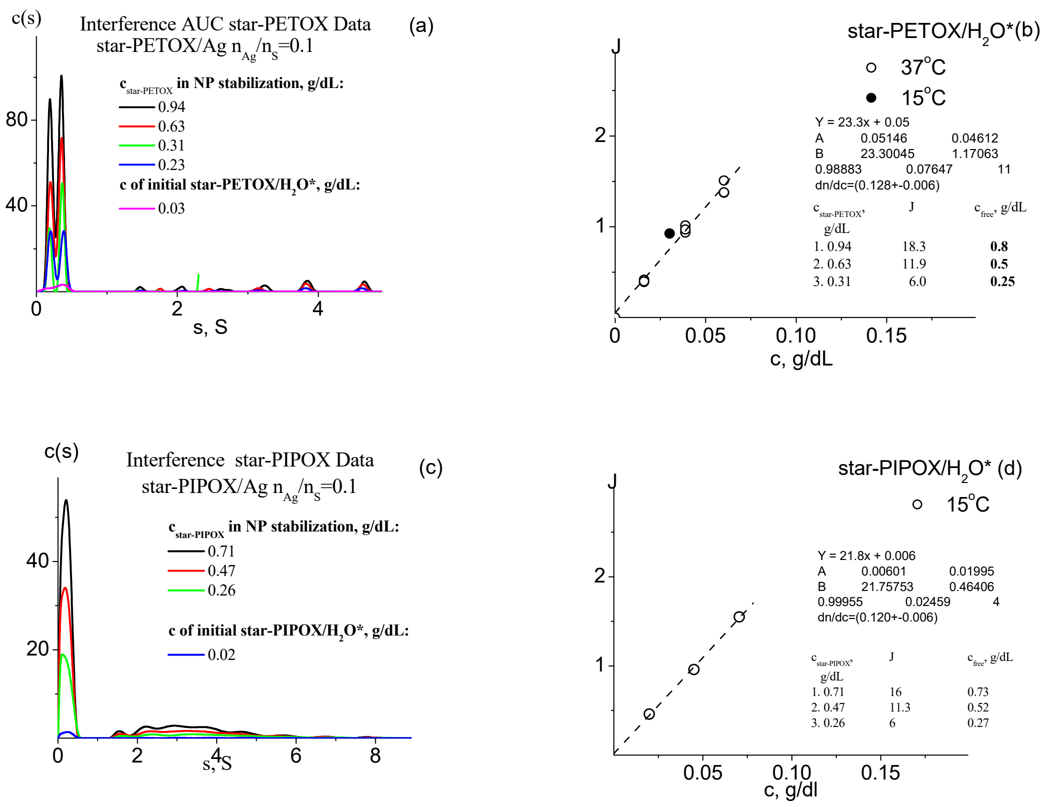

Figure S6. (**a**,**c**). The sedimentation coefficient distributions obtained inhere for the initial macromolecules star-PETOX(a) and star-PIPOX(c) while studying NP solutions with "differential sedimentation" approach and previously (*) obtained data on studying H_2O solution of initial star-PETOX(b) and star-PIPOX(d) systems [1]. The evaluation of "current" concentration of "free" macromolecules in silver NP solutions (**b**,**d**) were made based on the analysis of loading concentration parameter (J) reliably determined in [1]. Based on the estimations in average (90 ± 10)% of macromolecules are found "free" in NP solutions. The absolute value of the experimental error is mostly caused by the mismatch of the concentration ranges of studying initial macromolecules (where the calibration of J(c) is made) and the Ag NP solutions.

Table S1. Hydrodynami characteristics and molar mass of star-PETOX, star-PIPOX and its complexes with Ag nanoparticles.

Sample	c (g/dl)	Evaluation of c (g/dl)	*s* (S)	$(f/f_{sph})_0$	R_{sf} (nm)	R_{DLS} (nm)	$D_{DLS} \times 10^7$ (cm²/s)	M_{sD} (g/mol)
				star-PETOX/Ag^0 n_{Ag}/n_S=0.1				
NP1	0.94<br>0.63<br>0.31<br>0.23	0.8<br>0.5<br>0.25<br>-	12750<br>12610<br>13260<br>12100<br><12700>	1	<50><br>(30-70)**	58.4	0.32	1.3E9
NP2	0.94	0.8	600	1	<10><br>(6-14)**	7.9	2.37	8.1E6
star-PETOX			0.31	1.49*	~1-2	-	-	7.7E3*
				star-PIPOX/Ag^0 n_{Ag}/n_S=0.1				
NP	0.71<br>0.47<br>0.26<br>0.66<br>0.24	0.73<br>0.52<br>0.27	11500<br>11600<br>11200<br>12300<br>11700<br><11700>	1	<45><br>(30-60)**	59.2	0.31	1.2E9
star-PIPOX			0.21	1.42*	~1-2	-	-	4.6E3*

* Molar masses and $(f/f_{sph})_0$ values of individual star-PETOX and star-PIPOX macromolecules, obtained in tetrahydrofuran solution, where aggregation is absent [1] ** the presented data range is obtained in acordance with experimental error of the partial specific volume.

SANS data

SLD calculation. In small-angle scattering experiments, scattering occurs due to the contrast in neutron scattering length densities (SLD). In the two-phase system approach, the contrast is described by the following formula:

$$\Delta\rho = |\rho_1 - \rho_2| \quad \text{(S1)}$$

where ρ_1 and ρ_2 are the SLDs of the first and second phases forming the object under study, respectively. The SLD value, in turn, can be calculated by the formula:

$$\rho = N\sum_i b_i = (\delta N_A/M)\sum_i b_i \quad \text{(S2)}$$

where δ is the volume density of the object, N is the volume concentration of scattering centers, M is the relative molecular mass, N_A is the Avogadro's number, b_i is the scattering length of the ith nucleus in the molecule. The length densities of coherent scattering ρ_{coh} for the phases that constituted the objects under investigation are listed in Table S2.

Table S2. The values of scattering length density [a] ($\rho/10^{-6}$Å^{-2}) for neutrons in the systems under investigation.

System	Polymer			Ag^0 NPs		D_2O	
	Stabilizer	δ (g·cm^{-3})	n^0	Density (g·cm^{-3})	n^0	Density (g·cm^{-3})	n^0
S1	star-PETOX	1	0.9	–	–	1.1	6.3
S2	star-PETOX	1	0.9	10.5	3.5		
S3	star-PIPOX	1	0.9				

Notes: [a] Scattering length density was calculated with the use of a calculator https://www.ncnr.nist.gov/resources/activation/.

Generalized Guinier/Porod approximation. For an approximation of the SANS data, we used an empirical model [2], in which multiple Guinier and Porod regions can be identified, as:

$$I(Q) = \begin{cases} G\,Q^{-s} exp\left(-\dfrac{R_g^2 Q^2}{3-s}\right) & Q \le Q_1 \\ DQ^{-m} & Q \ge Q_1 \end{cases} \quad \text{(S3)}$$

where G is the exponential (Guinier) prefactor, s is a dimension variable, R_g is a Radius of gyration, m is a Porod exponent.

The prefactor D, and together with the quantity Q_1, are obtained from the continuity conditions of the Guinier and Porod terms as well as of their derivatives. They can be written explicitly as:

$$Q_1 = \frac{1}{R_g}\sqrt{(m-s)(3-s)/2}$$

and respectively:

$$D = G\,exp\left(-\frac{R_g^2 Q^2}{3-s}\right) Q_1^{m-s}.$$

The first term in Equation S3 is the generalized Guinier law, which is determining method for calculating the sizes of scattering objects of any shape in the region of small Q ($QR_g < 1$). For 3D globular objects (such as spheres), s = 0 and one recovers the standard Guinier formula. For 2D symmetry (such as for rods) s = 1, and for 1D symmetry (such as for lamellae or platelets) s = 2 [3,4]. The fractal dimension, D^m, and equilibrium structure qualities were evaluated from a relatively larger Q region using the Porod low—the second term in Equation S3. The fitting results of the experimental SANS data (Figure S7) are collected in Table S2.

Generalized Guinier/Porod approximation. The observed scattering intensity was treated in the frame of the unified global scattering function for three structural levels, and for the general case can be written as [5]:

$$I(Q) = \sum_{i=1}^{2}[G_i \exp(\frac{-Q^2R_{gi}^2}{3}) + B_i \exp(\frac{-Q^2R_{gi+1}^2}{3})(1/Q_i^*)^n] + C \tag{S4}$$

where i is a variable from 1 to 2 and denotes the structural levels described above. Each structural level forming a supramolecular organization (SMO), larger as the index i in Equation (S4) increases, is composed wholly of the previous level [5]. Factor G_i is directly proportional to the product of the number of mass-fractal groups in the scattering volume and the average neutron scattering length density on them, and factor B_i depends on the fractal dimension of the system. R_{gi} is the radius of gyration, n_i is the Porod exponent, and C is the residual incoherent background. Variables Q and Q^* are renormalized using the error function, erf (x), in the power laws as follows:

$$Q_i^* = Q/[\mathrm{erf}\,(kQR_{g,i}/\sqrt{6})]^3 \tag{S5}$$

This procedure allows one correctly to describe the behavior of the neutron scattering intensity, $I(Q)$, in the "intermediate" interval between QR_g < 1 (Guinier approximation) and QR_g >> 1 (asymptotic Q^{-n}). The experimental data are approximated using the method of least squares. The results of this analysis are presented in Figure 5 (solid curves) and Table 3 in main text.

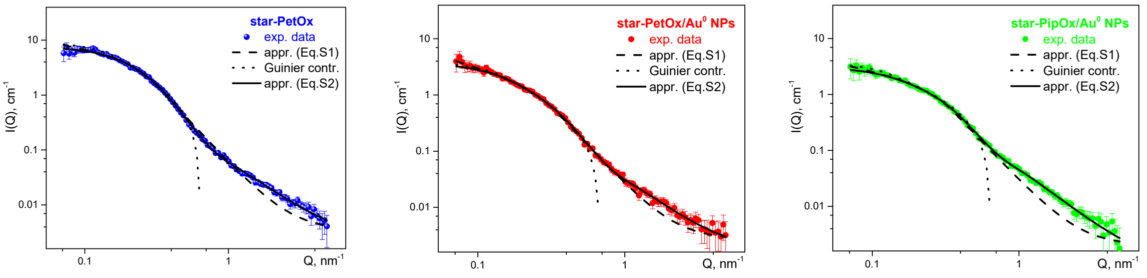

a b c

Figure S7. SANS data (dots) with approximations by Equation S3 (solid lines) with a contribution of the Guinier part (dot lines) and by Equation S2 (dash lines) for star-PETOX 6 wt % in D_2O (**a**), star-PETOX/Au^0 NPs 6/1 wt % in D_2O (**b**) and star-PIPOX/Au^0 NPs 6/1 wt % in D_2O (**c**).

Table S3. Fitting results of the SANS data, presented in Figure S2, by empirical model Equation S3.

complex	R_g (nm)	s	n
star-PETOX	7.9 ± 0.1	0.0	2.91 ± 0.01
star-PETOX/Ag^0 NPs	8.5 ± 0.2	0.0038	2.81 ± 0.03
star-PIPOX/Ag^0 NPs	8.0 ± 0.2	0.0	2.78 + 0.02

References

1. Lezov, A.A.; Gubarev, A.S.; Podsevalnikova, A.N.; Senchukova, A.S.; Lebedeva, E.V.; Dudkina, M.M.; Tenkovtsev, A.V.; Nekrasova, T.N.; Andreeva, L.N.; Smyslov, R.Y.; et al. Temperature-responsive star-shaped poly(2-ethyl-2-oxazoline) and poly(2-isopropyl-2-oxazoline) with central thiacalix[4]arene fragments: structure and properties in solutions. *Colloid Polym Sci* **2019**, *297*, 285–296.

2. Hammouda, B. A new Guinier–Porod model. *Journal of Applied Crystallography* **2010**, *43*, 716–719.

3. Hjelm, R.P.; Thiyagaragan, P.; Sivia, D.S.; Lindner, P.; Alkan, H.; Schwahn, D. Small-angle neutron scattering from aqueous mixed colloids of lecithin and bile salt. In Proceedings of the Trends

in Colloid and Interface Science IV; Zulauf, M., Lindner, P., Terech, P., Eds.; Steinkopff: Darmstadt, 1990; pp. 225–231.

4. Hjelm, R.P.; Thiyagarajan, P.; Alkan, H. A small-angle neutron scattering study of the effects of dilution on particle morphology in mixtures of glycocholate and lecithin. *Journal of Applied Crystallography* **1988**, *21*, 858–863.
5. Beaucage, G. 2.14 - Combined Small-Angle Scattering for Characterization of Hierarchically Structured Polymer Systems over Nano-to-Micron Meter: Part II Theory. In *Polymer Science: A Comprehensive Reference*; Matyjaszewski, K., Möller, M., Eds.; Elsevier: Amsterdam, 2012; pp. 399–409 ISBN 978-0-08-087862-1.
